# Diagnosis of Isolated Cleft of the Anterior Mitral Leaflet in a Dog: A Case Study Using Real-Time Three-Dimensional Echocardiography

**DOI:** 10.1155/2021/6610526

**Published:** 2021-01-26

**Authors:** Ryuji Araki, Koji Iwanaga, Kazunori Ueda, Mitsuhiro Isaka

**Affiliations:** ^1^Yokohama Yamate Dog & Cat Medical Center, 27-4 Kashiwaba Naka-ku Yokohama, Kanagawa, Japan 231-0866; ^2^Tokyo Veterinary Cardiology Center, 8-9-12 Izumi Building 2F, Fukazawa, Setagaya, Tokyo 158-0081, Japan; ^3^Department of Small Animal Clinical Sciences, School of Veterinary Medicine, Rakuno Gakuen University, 582 Bunkyodai-Midorimachi, Ebetsu, Hokkaido 069-8501, Japan

## Abstract

Isolated cleft of the anterior mitral leaflet (ICAML) in dogs without a septal defect is a rare pathological condition. Until now, only one paper has contributed to the detailed understanding of canine ICAML. Reports have confirmed that 3-dimensional echocardiography (3-DE) is a simple and fast imaging technique that is useful for the diagnosis of ICAML and morphological evaluation of the mitral valve in humans. However, to our knowledge, no studies have provided details about the effectiveness of 3-DE in ICAML diagnosis in dogs. Thus, we aimed to determine the usefulness of a diagnostic technique using 3-DE in a 2-year-old Cavalier King Charles Spaniel with ICAML that exhibited mild mitral valve regurgitation. ICAML was initially assessed by transthoracic two-dimensional echocardiography. A diagnosis of congenital mitral regurgitation due to ICAML and understanding of the morphological structure of the valve was established based on the 3-DE findings.

## 1. Introduction

Isolated cleft of the anterior mitral leaflet (ICAML, not associated with atrioventricular septal defect) is a very rare cause of congenital mitral regurgitation (MR) in humans [1–3]. Only one case has been reported on the occurrence of ICAML in dogs [4]. Until now, a clear prognosis of the disease and the need for long-term comprehensive care have not been clarified. However, there are some studies in humans that have explored the application value of 3-dimensional echocardiography (3-DE) in the diagnosis of ICAML and morphological evaluation of the mitral valve in humans [5, 6]. Therefore, this case study is aimed at exploring the application value of 3-DE in the diagnosis of ICAML in dogs.

## 2. Case Presentation

The patient was a 2-year-old castrated male Cavalier King Charles Spaniel that weighed 8.2 kg. The patient had a left ventricular systolic murmur confirmed at another hospital at 4 months of age. MR was confirmed using color-flow Doppler instead of two-dimensional echocardiography (2-DE). The patient was diagnosed with MR due to mitral valve dysplasia. The patient had been receiving temocapril hydrochloride (0.12 mg/kg/SID) and pimobendan (0.3 mg/kg/BID) from the age of 10 months. The patient came to our hospital for cardiac scrutiny.

On presentation to the hospital, the animal showed a heart rate of 147 beats per minute, a blood pressure of 117/80 mmHg, and oxygen saturation of 96%. A grade IV/VI left apical systolic murmur was heard on auscultation. There were no symptoms such as coughing or exercise intolerance, and no abnormal findings were found in blood cell counts or biochemical tests. Normal levels of cardiac biomarkers, namely, N-terminal probrain natriuretic peptide (655 pmol/dL; standard value: <900 pmol/dL), atrial natriuretic peptide (14.5 pg/mL; standard value: 8.6-105.8 pg/mL), and cardiac troponin I (0.012 ng/mL; standard value: <0.129 ng/mL), were observed.

**Figure 1:**
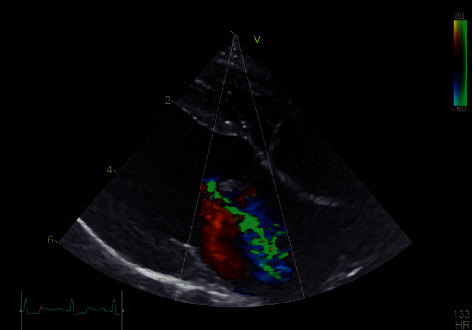
Color-flow Doppler echocardiogram obtained in the right parasternal long-axis view during systole. Mitral valve regurgitation is visible.

**Figure 2:**
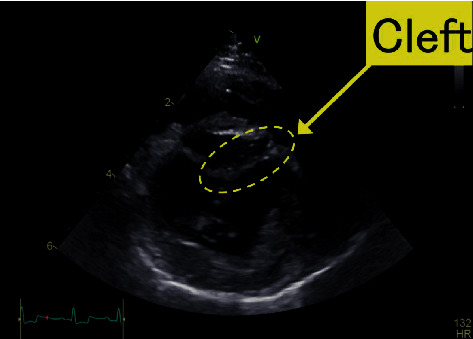
Two-dimensional echocardiography image. In the right parasternal short-axis view at the level of the mitral valve, a cleft is observed in the A2 segment of the anterior leaflet of the mitral valve.

**Figure 3:**
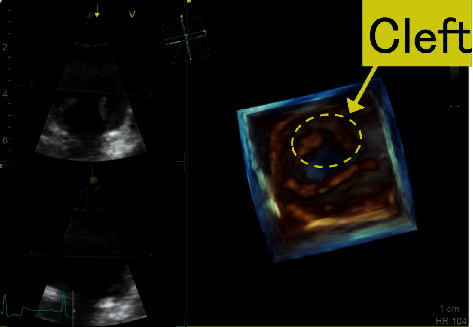
Three-dimensional echocardiographic image of the right parasternal short-axis view at the level of the mitral valve. A cleft of the anterior leaflet of the mitral valve can be clearly confirmed. The cleft is a V shape, with a maximum diameter of 2.1 cm and a maximum of crack by cleft width of 0.8 cm.

Thoracic radiography revealed a vertebral heart scale of 10.1 v (standard value: 10.6 ± 0.5 v) with no cardiac enlargement. No abnormal findings were found in the lung parenchyma or chest cavity, and there was no pulmonary vascular shadow. Electrocardiography revealed a sinus rhythm, and the electrical axis was 58°. Real-time 2-DE was performed using the ultrasound diagnostic equipment of Vivid E 95 (GE Healthcare, Japan). MR was observed on a left parasternal long-axis view using color-flow Doppler echocardiography (Figure 1). A cleft of the anterior leaflet of the mitral valve was observed from the left ventricular short-axis view at the level of the mitral valve (Figure 2). There were no defects in the atrium or ventricular septum. To obtain a detailed view of the mitral morphology, real-time three-dimensional (3-D) echocardiography (3-DE) was performed using a 4v probe. It directly showed the 3-D structure of the mitral valve cleft (MVC) in multiple views, revealing the position, shape, longitudinal diameter, and width, as well as the spatial position between the chordae tendineae surrounding the MVC and the aortic valve. The examination also revealed a maximum diameter of 2.1 cm, a maximum of crack by cleft width of 0.8 cm, and a V shape (Figure 3).

The blood flow velocity wave was measured by the continuous-wave Doppler method, which showed a maximum speed of 5.01 m/s and a maximum pressure difference of 100 mmHg in the mitral valve regurgitant flow velocity waveform. In the left ventricular inflow velocity waveform, the E wave and A wave were 0.63 and 0.37 m/s, respectively. Morphological evaluation was performed using the left parasternal short-axis view. This revealed a left atrium-to-aorta ratio of 1.56 at the aortic valve level (standard value: <1.6), a left ventricular end-diastolic diameter of 33.0 mm at the papillary muscle level, and the normalized left ventricular internal dimension in diastole (1.67; standard value: <1.7). On the basis of these findings, we judged that the severity of the MR was mild.

The examinations led to a diagnosis of congenital MR due to ICAML. Although organic cardiac abnormality was observed, there was no heart enlargement (mitral valve disease was classified as Stage B1 per the American College of Veterinary Internal Medicine classification). Following discussion with the owner, treatment with oral temocapril hydrochloride and pimobendan, as prescribed from other hospitals, was continued. The symptoms related to the cardiac condition remained unchanged and did not worsen without treatment until the dog died of lymphocytic leukemia.

## 3. Discussion

Although anterior MVC can be encountered within the spectrum of atrioventricular septal defects (AVSD), it is rare for them to be diagnosed as an isolated finding in dogs. ICAML is assumed to be a partial defect of the endocardial bed [1–3]. In human medicine, the median duration from birth to diagnosis is reported to be 2 or 5 years [1, 3]. In addition, among 2,177 patients with congenital heart disease, 22 (1%) were reported to have isolated cleft mitral valve, and 5 cases were not related to diseases such as AVSD [3]. Depending on the severity of MR, surgical treatment such as direct suturing of the valve is indicated, and its prognosis has been proven to be good [6]. Complete diagnosis and structural evaluation of congenital mitral valve malformations using real-time 2-DE may be difficult because of the complicated 3-D structure. Better morphometric and functional evaluations can be obtained with real-time 3-DE [7, 8]. It is important to measure the position and size of MVC in preparation for surgery, and real-time 3-DE is reportedly an important tool for cleft diagnosis and morphological mitral valve evaluation in humans [5, 6]. In our case, although ICAML was observed on real-time 2-DE images, evaluation of the morphological structure, such as the exact position of the cleft and the size and thickness of the valve, was difficult. In terms of spatial visualization of MVC, real-time 3-DE enabled us to obtain information that could not be elucidated by real-time 2-DE, as observed in human medicine. Although MVC associated with AVSD in dogs has been reported [9], preoperative evaluation using real-time 3-DE has not been performed. Detailed mitral valve assessments using real-time 3-DE will be very useful for mitral valve surgery in dogs with or without AVSD.

The occurrence of ICAML in canines is rare and has only been reported once [4]. Thus, the present case is a very rare clinical case. In a case report by Otoni and Abbott, MVC-induced MR and left ventricular dynamic outflow tract obstruction were seen, but the symptoms related to the cardiac condition in this animal did not worsen until it died of lymphocytic leukemia, so they remained untreated [4]. Our patient did not show dynamic left ventricular outflow stenosis due to the cleft; however, symptoms could be worsened in the presence of acquired factors such as myxoma-like degeneration. Further studies involving careful follow-up with surgical treatment are required. Overall, this case study suggests that real-time 3-DE may be useful for the detailed and accurate diagnosis of ICAML in dogs.
